# Patients’ views on health promotion and disease prevention services provided by healthcare workers in a South African tertiary hospital

**DOI:** 10.1186/s12913-023-09351-5

**Published:** 2023-04-15

**Authors:** Herbert I. Melariri, Chester Kalinda, Moses J. Chimbari

**Affiliations:** 1 College of Health Sciences, Disipline of Public Health Medicine, University of KwaZulu-Natal, Howard College Campus, Durban, 4041 South Africa; 2 Eastern Cape Department of Health, Port Elizabeth Provincial Hospital, Buckingham Rd, Mount Croix, Gqeberha, 6001 South Africa; 3 University of Global Health Equity (UGHE), Bill and Joyce Cummings Institute of Global Health, PO Box 6955, Kigali, 20093 Rwanda; 4 Great Zimbabwe University, PO Box 1235, Masvingo, Zimbabwe

**Keywords:** Health Promotion, Disease prevention, Patients’ views, Healthcare workers

## Abstract

**Background:**

Patients’ views and experiences in healthcare institutions provide a means of assessing the quality of services patients receive from healthcare workers (HCWs). However, the views of patients on the health promotion (HP) and disease prevention (DP) services offered by HCWs and the delivery mode have not been adequately studied.

**Aim:**

This study assessed the views of patients on HP and DP services provided by various categories of HCWs.

**Setting:**

The study was conducted at a tertiary hospital in the Nelson Mandela Bay Municipality, South Africa.

**Method:**

An exploratory cross-sectional study was conducted among 500 patients. The questionnaire elicited responses from patients regarding the HP and DP services received from the different cadres of HCWs at three different admission phases: pre-admission phase (PAP), admission phase (ADP), and post-admission phase (POP). Descriptive, bivariate, and multivariate analysis was conducted.

**Results:**

In the PAP, most patients (83.33%, n = 5; 87.85%, n = 217; and 76.14%, n = 150) seen by the rehabilitation health workers, medical doctors, and nurses respectively were empowered to manage their health. Patients attended to by nurses were 0.45 (95% CI 0.27–0.74) times less likely than those attended to by medical doctors to receive information that that will help them address the physical and environmental needs. In the ADP, patients attended to by nurses were less likely, compared to those attended to by medical doctors to be empowered to have good control over their health. In the POP, patients attended to by nurses are more likely to have their health behaviours change for better compared to those not seen by any HCW.

**Conclusion:**

Patients attending tertiary hospital received greater HP and DP services during the PAP and ADP of patient care. Greatest influence for behavioural change of patients on HP and DP were achieved from the medical doctors, nurses and rehabilitation service staff. Improving structural factors may prove beneficial in enhancing patients’ experience from all HCW groups and phases of patient care.

## Introduction

In addition to clinical services, patients receive HP and DP services [1, 2] from HCWs within and outside the healthcare facilities. These HP and DP services have improved health outcomes among patients, reduced disease burden, boosted cost-effectiveness, and improved patients’ experiences [3, 4]. Assessing patients’ views and perceptions is a vital tool in understanding how well healthcare services are delivered and received [5–7] and help to identify practical ways to enhance service delivery.

Health promotion refers to the process of enabling people to increase control over, and to improve their health [8]. Disease prevention on the other hand, describes measures to reduce the occurrence of risk factors, prevent the occurrence of disease, to arrest its prgress and reduce its consequences once established [8]. In South Africa, there is reduced coordinated HP and DP training for medical doctors compared to nurses [9].

Patients are key stakeholders in the healthcare system [10]. With an increasing focus on the quality of services delivered to patients [11], they are now more knowledgeable of their health conditions [12–14], know their rights [15], and freely convey their expectations concerning various healthcare services rendered to them by HCWs [16]. Previous studies have evaluated healthcare services in health institutions to measure, monitor and assess patients’ views on the health care services received from HCWs, including medical doctors, nurses, and rehabilitation service staff [7, 17]. According to Berger et al. [18], patient feedback is one of the major impact assessment indicators for service improvement and intervention. Berger et al. [18] described three forms of patient feedback: voluntary events, patients surveys, and informal feedback. In voluntary events, patients log complaints through available media such as customer portals, telephonic [19, 20] or email communication [21], or social media platforms. The feedback can also be initiated by the institution through periodic surveys done telephonically or issued to patients to complete. Informally, patients can also give feedback to HCWs verbally.

Although the literature on patients’ view of HP and DP services are limited, there is evidence suggesting that patients are distinctively positioned to guide HCWs on the quality of services they deliver. In the United States, most patients agreed that HCWs should be role models of behavioural change to them [22]. In France, Pinar et al. [23], while evaluating patient satisfaction during the COVID-19 pandemic suggested that patients who met their doctors for the first time were more likely to be satisfied. Reza et al. [24] demonstrated in a satisfaction survey, that the waiting time of patients at different service arms of the clinics influenced their overall satisfaction. A study by Freeman et al. [25] concluded that HP in South Africa, a country experiencing resource constraints affecting public healthcare service delivery, had great potentials to improve the cost-effectiveness of health outcomes. Although these authors assessed the views of HP practitioners, they did not assess the views of patients served. In addition, earlier studies focusing on HP or DP service assessments were conducted in countries with similar resource-constrained settings focusing on aspects such as nutrition promotion programs and promotion of physical activity in schools [26–28]. However, there is a dearth of information on studies evaluating the views of patients on HP and DP services rendered by HCWs in sub-Saharan countries, including South Africa.

In recent years, tertiary hospitals in the Nelson Mandela Bay Municipality, South Africa, have made some progress in HP and DP services to patients [29]. However, this progress is restricted as individuals and specific HCW groups work in silos. To improve the quality of HP and DP services delivered to patients, the extent of services rendered by different HCW groups should be evaluated at this healthcare level with considerations of the views of patients. This study, therefore, was conducted to assess the views of patients regarding HP and DP services they received from HCWs.

## Methods

### Study design and sample

An exploratory cross-sectional study was conducted among patients referred to the outpatient and in-patient departments at a tertiary hospital in Nelson Mandela Bay Municipality, Eastern Cape Province in South Africa. The Nelson Mandela Bay Municipality is an important economic hub in South Africa as well as a reference in healthcare services. The tertiary hospital was selected as the setting for this study because it serves a catchment population of about 1.6 million, mainly from the Eastern Cape province. The sample population comprised all adult outpatients and inpatients of the hospital. We did not include critically ill patients and patients in intensive care unit for ethical reasons. Including these patients would have increased their stress and probably made their conditions worse.

Participants were selected using a homogenous purposive sampling strategy [30]. This strategy was used to include only patients that share the common characteristic of having been fully attended to by HCWs, either in the current or previous visits to the hospital. A total of 500 patients agreed to participate in the study by signing a written consent form. We purposively selected study participants because we wanted a sample size that was as large as possible. Since different patients were present on different days in the hospital, the study was conducted over three months to offer an equal chance for eligible participants to partake.

### Survey instrument

Data collection was achieved using a structured questionnaire herein referred to as Health Promotion Provision Assessment (HPPA) questionnaire. The major advantage of the HPPA is in it’s higher response rates as it is designed for easy response, while it’s main drawback is on the time taken by the fieldworkers to interview every participant.The HPPA comprised two sections; section A had three items where the participants could indicate which HCW cadre offered certain HP and DP services while section B contained eight items focusing on patients’ satisfaction and empowerment. The HCW cadres included medical doctors, nurses, rehabilitation health workers, dieticians, and social workers. The items in section B were measured on a four-point Likert scale ranging from 1 (strongly disagree) to 4 (strongly agree). Both sections have Cronbach’s alpha of 0.777 and 0.783 respectively. Data collection was conducted between January to March 2020 by trained field workers. The field workers were graduate students.

### Statistical analysis

Data was captured in Microsoft Excel 2016 and imported to StataIC 15 (Stata Statistical Software: Release 15. College Station, TX: StataCorp LLC) where data cleaning and analysis was done. Descriptive statistics were used to summarize the HP and DP services provided by different HCWs. Analysis was conducted for the three-tiered phase outcome measures comprising: pre-admission, admission, and post-admission. Pre-admission phase refers to the period of out-patient consultations and period before getting a bed in the hospital ward. Admission phase refers to the period in which patient is admitted in hospital and treated as in-patient. Post admission refers to period after a patient has been discharged from hospital and is at home. Associations between the outcome and predictor variables were assessed in a bivariate analysis using a Pearson chi-square test otherwise, a Fisher’s test was done where the same frequencies were small. This was done with phase outcome measures (pre-admission, admission, and post-admission). At the pre-admission phase, analysis focused on determining which HCW group properly attended to patients’ HP and DP needs. During the admission phase, the HCW group that attended to the patients at this stage was considered as the predictor variables, while the HCW group that followed the health progress of patients after discharge was considered for the post-admission phase. Predictor variables associated with the response variable in the bivariate analysis were used in a multinomial regression model. The Hosmer & Lemeshow test was used in checking the models’ goodness of fit [31].

All methods ensured adherence to the following guidelines and regulations: (1) Valid scientific design and conduct of the study were ensured; (2) Potential harms were prevented; (3) No participant was made to bear more than his/her fair share of the burden of participation in the study; (4) Protection of research participants’ privacy and confidentiality was ensured; and (5) Participants were entitled to choose freely whether to participate in the research, and to make decisions based on an adequate understanding of what the study entails [32].

## Results

### Pre-admission phase (PAP)

In the pre-admission phase, significant associations between HP/DP practices and the different cadres of HCWs were identified on five out of eight variables under consideration (Table 1). Statistically significant variables included - information helping patients address their physical and environmental needs (*p* < 0.001), patients’ being empowered to manage their health (*p* = 0.001), patients’ satisfaction with HP services (*p* = 0.002), patients’ health behaviour changing for better (*p* = 0.027), and patients being empowered to have good control over their health (*p* = 0.011). A 100% response rate was recorded by patients who were educated by rehabilitation health workers on the importance of treatment compliance. When compared across the various HCW cadres, rehabilitation health workers empowered 83.33% of their patients to have control over their health, 87.85% by medical doctors, and 76.14% by nurses. Regarding influencing patients to change their health behaviour for better, 33.33% of patients were not attended to by the rehabilitation health workers; 20.24% were not attended to by the medical doctors; and 25.00% were not attended to by the nurses.

**Table 1:** Bivariate analysis between outcome variable (Attending HCW at Pre-Admission Phase) and predictor variables

Predictors	Responses	Attending HCW at pre-Admission Phase (PAP) (Frequency, %)				*p-value*
		Rehab	Doctors	Nurses	None	
Educated on importance of treatment compliance	Not attended	0	27(10.76%)	17(8.46%)	6(17.65%)	0.351*
	Attended to	6(100%)	224(89.24%)	184(91.54%)	28(82.35%)	
Educated on the benefits of physical exercise and fitness	Not attended	2(33.33%)	73(28.97%)	66(32.84%)	17(50%)	0.098*
	Attended to	4(66.67%)	179(71.03%)	135(67.16%)	17(50%)	
Received Information concerning preventable diseases	Not attended	3(50.00%)	135(53.78%)	99(49.25%)	19(55.88%)	0.750*
	Attended to	3(50.00%)	116(46.22%)	102(50.75%)	15(44.12%)	
Addressed physical and environmental needs	Not attended	1(16.67%)	42(16.80%)	65(32.66%)	14(42.42%)	0.000*
	Attended to	5(83.33%)	208(83.20%)	134(67.34%)	19(57.58%)	
Empowered to manage health	Not attended	1(16.67%)	30(12.15%)	47(23.86%)	11(34.38%)	0.001*
	Attended to	5(83.33%)	217(87.85%)	150(76.14%)	21(65.62%)	
Satisfied with HP services	Not attended	1(16.67%)	37(14.86%)	50(25.13%)	13(40.63%)	0.002*
	Attended to	5(83.33%)	212(85.14%)	149(74.87%)	19(59.37%)	
Health behaviour changed for better	Not attended	2(33.33%)	50(20.24%)	49(25.00%)	14(43.75%)	0.026*
	Attended to	4(66.67%)	197(79.76%)	147(75.00%)	18(56.25%)	
Good control over health	Not attended	2(33.33%)	32(12.96%)	35(17.77%)	11(34.38%)	0.011*
	Attended to	4(66.67%)	215(87.04%)	162(82.23%)	21(65.62%)	

HP, Health Promotion; Rehab, Rehabilitation health workers; PAP, Pre-admission phase; *Fishers exact test was used because some frequencies were less than 10

In the final model of the multinomial regression analysis, three HP/DP variables were significantly associated with nurses’ practice, and none was identified for rehabilitation health workers (Table 2). The analysis showed that patients were 1.54 (95% CI: 1.03–2.30) times as likely to receive information about preventable diseases from nurses as they were from medical doctors. The results further showed that patients were 32% (RR: 0.68; 95% CI: 0.33–0.99) less likely to be empowered by nurses than medical doctors to manage their health. In addition, the results also showed that patients were 64% (RR: 0.36; 95% CI: 0.14–0.88) and 61% (RR: 0.39; 95% CI: 0.16–0.97) less likely to be addressed on physical and environmental needs and empowered to manage their health, respectively by none of the health workers compared to doctors.

**Table 2:** Multinomial regression model analysis relating HP and DP services and HCW groups in the PAP

Predictors	Relative Risk Ratio	*p*-value	95% Confidence Interval
**Rehab**			
Received Information concerning preventable diseases	1.12	0.887	0.21–5.98
Addressed physical and environmental needs	1.06	0.959	0.09–11.63
Empowered to manage health	0.67	0.738	0.07–6.92
**Nurses**			
Received Information concerning preventable diseases	**1.54**	**0.035**	**1.03–2.30**
Addressed physical and environmental needs	**0.45**	**0.002**	**0.27–0.74**
Empowered to manage health	**0.68**	**0.047**	**0.33–0.99**
**None**			
Received Information concerning preventable diseases	1.40	0.405	0.63–3.10
Addressed physical and environmental needs	**0.36**	**0.026**	**0.14–0.88**
Empowered to manage health	**0.39**	**0.043**	**0.16–0.97**

Doctors as Reference outcome; Rehab, Rehabilitation health workers

### Admission phase (ADP)

Bivariate analysis of the admission phase showed a 100% response from patients who were attended to by the dieticians (Table 3). The results shows that four out of the eight HP/DP variables were significantly associated with the various healthcare professional groups. The statistically significant variables elicited in this phase are – information addressing physical and environmental needs of patients (*p =* 0.045), empowerment of patients to manage their health (*p =* 0.000), patients health behaviour changed for better (*p <* 0.001), and empowering patients to have good control over health (*p =* 0.000). Regarding patients’ empowerment to have good control over their health, the results show that 100% of the dieticians’ and rehabilitation health workers’ patients were fully attended to. A total of 86.19%  and 73.56% of patients seeing the medical doctors and nurses respectively were also empowered to have good control over their health.

**Table 3:** Bivariate analysis between outcome variable (Attending HCW at Admission Phase) and predictor variables

Predictors	Responses	Attending HCW at Admission Phase (ADP) (Frequency, %)					*p-value*
		Dieticians	Rehab	Doctors	Nurses	None	
Educated on importance of treatment compliance	Not attended	0	0	37(10.08%)	10(11.11%)	4(25.00%)	0.228*
	Attended to	11(100%)	12(100%)	330(89. 92%)	80(88.89%)	12(75.00%)	
Educated on the benefits of physical exercise and fitness	Not attended	2(18.18%)	1(8.33%)	123(33.42%)	29(32.22%)	6(37.50%)	0.349*
	Attended to	9(81. 2%)	11(91.67%)	245(66.58%)	61(67.78%)	10(62.50%)	
Received Information concerning preventable diseases	Not Attended	4(36.36%)	5(41.67%)	196(53.41%)	43(47.78%)	10(62.50%)	0.528*
	Attended to	7(63.64%)	7(58.33%)	171(46.59%)	47(52.22%)	6(37.50%)	
Addressed physical and environmental needs	Not attended	2(18.18%)	1(8.33%)	83(22.87%)	33(36.67%)	5(31.25%)	0.048*
	Attended to	9(81.82%)	11(91.67%)	280(77.13%)	57(63.33%)	11(68.75%)	
Empowered to manage health	Not attended	0	0	57(15.79%)	29(32.95%)	6(37.50%)	0.000*
	Attended to	9(100%)	12(100%)	304(84.21%)	59(67.05%)	10(62.50%)	
Satisfied with HP services	Not attended	1(9.09%)	2(16.67%)	67(18.51%)	24(26.97%)	7(43.75%)	0.060*
	Attended to	10(90.91%)	10(83.33%)	295(81.49%)	65(73.03%)	9(56.25%)	
Health behaviour changed for better	Not attended	0	0	82(22.71%)	21(24.14%)	13(81.25%)	0.000*
	Attended to	9(100%)	12(100%)	279(77.29%)	66(75.86%)	3(18.75%)	
Good control over health	Not attended	0	0	50(13.81%)	23(26.44%)	8(50.00%)	0.000*
	Attended to	9(100%)	12(100%)	312(86.19%)	64(73.56%)	8(50.00%)	

HP, Health Promotion; ADP, Admission phase; • Fishers exact test was used because some frequencies were less than 10

The multivariate analysis of ADP (Table 4) revealed three statistically significant dimensions that were associated with the HCW group that attended to the patients. The analysis showed nurses were 57% (RR: 0.43; 95% CI: 0.24–0.78) and 54% (RR: 0.46; 95% CI: 0.22–0.95) less likely to empower patients to manage their health and take good control over health during admission, respectively compared to doctors. The result further showed that patients who were not attended to by any health worker were 0.09 (95% CI 0.02–0.37) times less likely to change their health behaviour for better when compared to patients attended to by medical doctors.

**Table 4:** Multinomial regression model analysis relating HP and DP services and HCW groups in ADP

Predictors	Relative Risk Ratio	*p*-value	95% Confidence Interval
**Dieticians**			
Empowered to manage health	0.63	0.150	0.33–0.74
Health behaviour changed for better	0.47	0.092	0.16–1.14
Good control over health	0.73	0.59	0.26–2.11
**Rehab**			
Empowered to manage health	0.53	0.20	0.20–1.42
Health behaviour changed for better	0.48	0.67	0.19–1.47
Good control over health	0.77	0.67	0.24–2.47
**Nurses**			
Empowered to manage health	**0.43**	**0.006**	**0.24–0.78**
Health behaviour changed for better	1.79	0.104	0.89 − 0.58
Good control over health	**0.46**	**0.036**	**0.22–0.95**
**None**			
Empowered to manage health	0.84	0.772	0.25–2.81
Health behaviour changed for better	**0.09**	**0.001**	**0.02–0.37**
Good control over health	0.58	0.388	0.17-2.00

Doctors as reference outcome; Rehab, Rehabilitation health workers

### Post Admission Phase (POP)

Results emanating from the bivariate analysis of the POP showed no statistically significant association between the HP/DP variables and the various HCW cadres. The results show a 100% response from patients who were attended to by rehabilitation health workers on the importance of treatment compliance (Table 5). The results further showed that 70% of patients attended to by rehabilitation health workers were educated on the benefits of physical exercise and fitness. Similarly, 60% and 75% of patients were respectively educated by the medical doctors and nurses on the benefits of physical exercise and fitness.

**Table 5:** Bivariate analysis between outcome variable (Which HCW group gave you a call post-admission?) and predictor variables

Predictors	Responses	Attending HCW at Post Admission Phase (POP) (Frequency, %)				*p-value*
		Rehab	Doctors	Nurses	None	
Educated on importance of treatment compliance	Not attended	0	1((4.00%)	5(20.83%)	45(10.51%)	0.244*
	Attended to	10(100%)	24(96.00%)	19(79.17%)	383(89.49%)	
Educated on the benefits of physical exercise and fitness	Not attended	3(30.00%)	10(40.00%)	6(25.00%)	140(32.63%)	0.737*
	Attended to	7(70.00%)	15(60.00%)	18(75.00%)	289(67.37%)	
Received Information concerning preventable diseases	Not attended	4(40.00%)	11(45.83%)	12(50.00%)	230(53.49%)	0.737*
	Attended to	6(60.00%)	13(54.17%)	12(50.00%)	200(46.51%)	
Addressed physical and environmental needs	Not attended	1(10.00%)	4(17.39%)	11(45.83%)	105(24.65%)	0.077*
	Attended to	9(90.00%)	19(82.61%)	13(54.17%)	321(75.35%)	
Empowered to manage health	Not attended	1(10.00%)	4(17.39)	8(36.36%)	77(18.25%)	0.186*
	Attended to	9(90.00%)	19(82.61%)	1463.64%)	345(81.75%)	
Satisfied with HP services	Not attended	1(10.00%)	7(29.17%)	7(31.82%)	84(19.76%)	0.287*
	Attended to	9(90.00%)	17(70.83%)	15(68.18%)	341(80.24%)	
Health behaviour changed for better	Not attended	1(10.00)	5(21.74%)	3(13.64%)	106(25.18%)	0.518*
	Attended to	9(90.00%)	18(78.26%)	19(86.36%)	315(74.82%)	
Good control over health	Not attended	1(10.00%)	3(13.04%)	7(31.82%)	70(16.59%)	0.293*
	Attended to	9(90.00%)	20(86.96%)	15(68.18%)	352(83.41%)	

HP, Health Promotion; HCW, Healthcare workers; POP, Post admission phase; *Fishers exact test was used because some frequencies were less than 10

Final model multivariate analysis of the POP identified three HP/DP variables that were significantly associated with nurses practice (Table 6). The analysis show that nurses in the POP were 3.13 (95%CI: 1.02–5.09) times more likely to influence patients change their health behaviour for better compared to patients not seen by any healthcare worker. The analysis further showed that patients who were attended to by nurses were 0.29 (95% CI 0.09–0.90) and 0.17 (95% CI 0.06–0.53) times less likely to be educated on importance of treatment compliance and empowered to have good control over health respectively, compared to patients attended to by no health worker.

**Table 6:** Multinomial regression model analysis relating HP/ DP services and HCW groups in POP

Predictors	Relative Risk Ratio	*p*-value	95% Confidence Interval
**Rehab**			
Educated on importance of treatment compliance	0.81	0.530	0.48–1.87
Health behaviour changed for better	2.56	0.425	0.25–25.93
Good control over health	0.95	0.967	0.09–9.72
**Doctors**			
Educated on importance of treatment compliance	2.36	0.411	0.30-18.36
Health behaviour changed for better	0.98	0.980	0.30–3.18
Good control over health	1.24	0.764	0.30–5.20
**Nurses**			
Educated on importance of treatment compliance	**0.29**	**0.031**	**0.09–0.90**
Health behaviour changed for better	**3.13**	**0.008**	**1.02–5.09**
Good control over health	**0.17**	**0.002**	**0.06–0.53**

None (no healthcare worker) as base outcome

## Discussion

The Health Promotion Provision assessment (HPPA) is a good tool for assessing the view of patients regarding the quality of health care services received from various groups of HCWs. We used it to assess the HP and DP services provided by the different HCW groups in three phases of patient care: pre-admission phase, admission phase, and post admission phase. Patients’ assessment of HCWs’ HP and DP performance were influenced by their experiences from the HCWs. Consistent with existing studies, our data show that patients received HP and DP services from HCWs mostly during the PAP and ADP [33–37], while the least of such services were received in the POP [38]. As observed in other studies [39–41], our study revealed that medical doctors had the greatest positive HP and DP influence on patients at both the PAP and ADP, while nurses influence was greatest at the POP .

In the PAP, our results showed that majority (greater than 50%) of patients seen by the various cadres of HCWs were adequately attended to across the statistically significant HP and DP services delivered. This finding may be related to the chosen study institution, in this case, a tertiary hospital. A tertiary hospital is a specialist centre [42] where patients with special needs beyond the care of the primary and secondary level hospitals are referred to. When patients with specific needs are seen by the appropriate specialists, chances are that they will receive the best care possible including HP and DP services. Although the rehabilitation health workers recorded a 100% response rate from patients regarding their role in educating patients on the importance of treatment compliance, this variable at PAP was not found to be statistically significant. Further in the PAP, greater number of patients were attended to by the medical doctors and nurses, and reasons for this may be due to the tailored needs of patients and a higher numerical staffing for these cadres of HCWs [29] compared to rehabilitation health workers in the hospital.

Again, in the PAP, patients’ satisfaction with HP services were 0.45 and 0.68 times lower by their interactions with nurses when compared to their interactions with medical doctors. Our findings corroborate with the results of Kalroozi, Dadgari, and Zareiyan [43] who reported 17.2% dissatisfaction of patients from nurses and only 8% dissatisfaction from medical doctors. The study of Karoozi et al. showed that high patients’ satisfaction with doctors was however demonstrated in the highly specialized wards of open-heart surgery. The current study, however, did not explore patients’ satisfaction at the specialized wards, and this warrants further exploration. Similarly, the study of Stump et al. 2019 [44] which showed that 90% of primary care physicians recommended HP and DP activities to their patients further supports our finding. Though our study’s finding shows that patients in the PAP were 1.12 times more likely to receive information concerning preventable diseases from the rehabilitation service staff compared to receiving it from medical doctors, this finding was not statistically significant, and search of the literature did not reveal a previous study to which this finding could be correlated. This finding can be explained in the light of the doctor – patient relationship which can be considered to be one of the most ethically significant dimensions of good medical care. It is during the interactions that constitute this relationship that information is shared, that choices get determined, that reassurances are provided, that decisions are made and, ultimately, that care is given, hence, the positive influence of medical doctors [45].

During the ADP, we found that patients’ empowerment to both manage and have good control over their health were 0.43 and 0.46 times less from their interactions with nurses when compared to their interactions with medical doctors. Although the contributions of all HCWs are strongly acknowledged and appreciated, during hospital admissions, patients believe that medical doctors have the final say regarding the quality of care they receive towards wellbeing. Further in the ADP, it was noted that patients ability to change their health behaviour for better was 1.79 times better from their interaction with nurses compared to medical doctors, this finding was however not statistically significant.

Results from the POP showed that nurses were more likely to influence better health behavioral change among patients compared to patients who were not attended to by any HCW. Further findings from the POP reveal that the influence from medical doctors, social workers, rehabilitation health workers, and dieticians was less likely to influence patients. This may be related to the minimal participation of these groups of HCWs in patients’ care once patients have been discharged. This minimal participation of certain groups of HCWs may be connected to the several challenges confronting the health department of the study area such as constrained human resources and infrastructure [46, 47]. At the POP, nurses were the only HCW group that positively influenced a HP/DP variable. This findings corroborate the study of Guzmán, Ferreira, and de Andrade (2020) which highlighted the important role nurses played through care networks in ensuring continuity of nursing services for discharged patients [48].

Although no study comparing HP and DP impact at the different phases was identified, most HP and DP services carried out at the primary healthcare centers are outpatient services (pre-admission phases). The WHO vision for primary healthcare in the 21st century recognizes HP and DP as a vital primary care responsibility in the delivery of comprehensive healthcare services [49]. Whilst some studies [50, 51] have attempted to explore the HP and DP services at primary healthcare, this study is distinctive in linking the HCWs HP and DP services at a tertiary level hospital with the accruing HP empowerment.

We also noted that two HP dimensions; ‘patients’ empowerment to manage their health’ and ‘patients’ empowerment to have good control over their health’ were the most recurrent variables. The former occurring twice in PAP and ADP, while the later dimension occurred twice in ADP and POP. The occurrence of both dimensions in the PAP and ADP may be related to most hospital outpatients and inpatients having risk factors [34] that are amenable to changes following their interactions with HCWs.

### Limitations and strengths

The study was conducted in only one tertiary hospital which draws patients from many hospitals within the region. Thus, we may not have accommodated some of the views of patients at the primary and secondary level hospitals. We have attempted to address this shortfall by considering patients that were referred to the hospital from the lower levels. Furthermore, the study did not include the specialized wards of the hospital and offers potential for future explorations. A major strength of this study is the comparison of patients’ views concerning the various HCW groups and the stratification of the various phases of patients care which makes it easier to identify which HCW group and care phase needs to be strengthened.

## Conclusion

This study presents evidence on patients’ views of HP and DP services offered by HCWs in a South African tertiary hospital context. The study revealed the complementarity of HCWs in delivering HP and DP services to patients, evidenced by the differential influences on patients by the different groups of HCW groups. While HP and DP services were delivered better to patients during the PAP and ADP, the least services were delivered during the POP, the impact of some HCWs were minimally noticed at all phases. The study revealed that nurses were less likely to empower patients to manage their health and take good control over health during admission, respectively compared to doctors. Our study expounds on essential elements that may assist HCWs and policymakers to predict and enhance quality within healthcare facilities. To improve on the quality of HP and DP services by HCWs, more attention needs to be paid to the POP and HCWs whose HP and DP services were identified to be less influential, and skills on key dimensions that potentiates outcome. Periodic trainings of HCWs on how to give effective HP and DP services using better communication methods is therefore recommended. Furthermore, it is important to conduct a periodic needs assessment to identify patients’ expectations and merge this with services from HCWs. Similar studies including details on participants’ demography should be conducted at the primary and secondary care levels. Such studies should also explore reasons why most HP and DP services were perceived to be given by certain HCWs and at certain phases.

## Data Availability

The datasets used and/or analyzed during the current study are available at the following link: 10.7910/DVN/9JXKCG.
